# A Novel Surgical Technique Using a Hockey Stick–Like Guided Knife to Go Through the Eyes of a Needle for Trigger Finger

**DOI:** 10.1016/j.jhsg.2023.10.007

**Published:** 2023-12-08

**Authors:** Saaya Amano, Yukio Mikami, Tetsushi Chikamoto, Kanzo Amano, Toshiaki Kanazawa, Nobuo Adachi

**Affiliations:** ∗ Department of Rehabilitation Medicine, Hiroshima University Hospital, Hiroshima, Japan; † Department of Orthopedic Surgery, Hiroshima Clinic, Hiroshima, Japan; ‡ Department of Orthopedic Surgery, Hiroshima University Hospital, Hiroshima, Japan

**Keywords:** Case report, Eyes of a needle technique, Percutaneous surgery, Special guided knife, Trigger finger

## Abstract

Trigger finger surgery is primarily managed with open surgery accompanied by 10–14 days of postoperative recovery, which may interrupt activities of daily living. In the past, we attempted to perform percutaneous surgery by inserting a hockey stick–shaped guide knife through a scalpel incision several millimeters long. Sometimes, we encounter difficult cases wherein triggering does not disappear despite repeated attempts to release the A1 pulley through the small incision, thus forcing us to extend the incision. As a result, the postoperative recovery is sometimes prolonged. We describe our experience using a novel percutaneous procedure in which a guide knife was inserted through one or two 20-gauge needle holes, instead of a scalpel skin incision, to release the A1 pulley. We describe a new method that minimizes skin and soft tissue damage and reliably shortens posttreatment recovery.

Trigger finger is one of the most frequently encountered hand disorders. It is characterized by snapping and locking caused by an imbalance between the sizes of the flexor tendon and tendon sheath.^1^^,^^2^ The condition affects activities of daily living, such as dressing or kitchen work, due to painful snapping when bending the fingers. Surgery and corticosteroid injections are the most common treatment options.

According to the Multidisciplinary Consensus Guidelines, surgery should be considered for the treatment of trigger finger in patients who do not improve after one to three steroid injections.^3^ Steroid injections may not remain effective for the mid or long term, although they are a cost-effective, minimally invasive treatment with a rapid posttreatment response. In contrast, surgery is invasive, but recurrence is rare. The main surgical procedures are open and percutaneous surgery. Open surgery using local anesthesia, a transverse incision, and nonresorbable sutures may be preferable to a percutaneous technique for treating trigger finger because it allows for a more meticulous inspection of the surgical area.^3^ However, the recurrence rates and complications associated with both surgical techniques are comparable. The greatest advantage of percutaneous surgery may be the small incision that allows for rapid postsurgical recovery and rehabilitation; however, this factor was not included in the aforementioned guidelines.^3^

At our clinic, in the past, we performed minimally invasive, safe, and reliable percutaneous surgery using a hockey stick–shaped guide knife (Y’s Guided Knife, Tsuda Medical Equipment Corp). Y’s Guided Knife is inserted through a scalpel incision of several millimeters and was developed by Yumoto.^4^ Y’s Guided Knife contains a set of two blades, one with a dull edge (guide section) and the other with a sharp edge (blade section), which were used in this study. The guide section of the Y’s Guided Knife is 4 mm long, and it is positioned outside of the outer curve, which is bent like a hockey stick to avoid damage to the skin and soft tissue (Fig. 1).^4^

**Figure 1:**
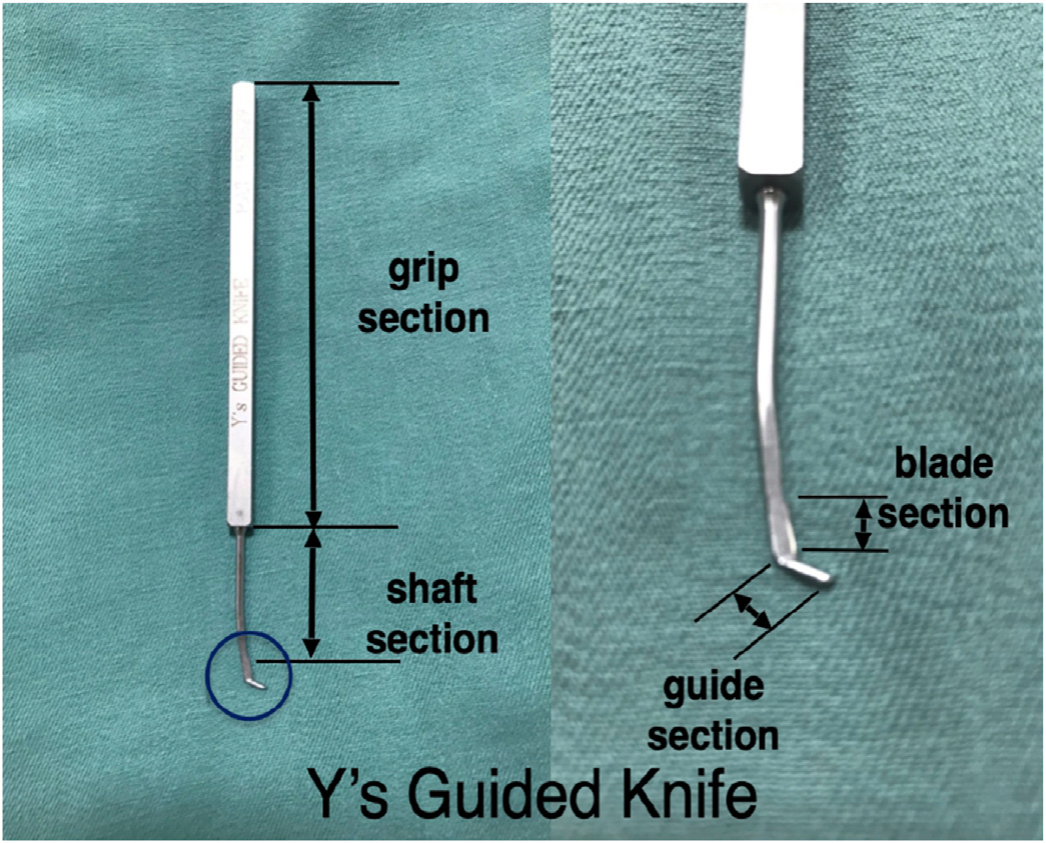
Y’s Guided Knife structure. Y’s Guided Knife has two similarly shaped blades, one with a dull edge and the other with a sharp edge. The right image shows an enlarged view of the blade.

However, it is difficult to completely release the A1 pulley with a single thrust cut unless the procedure is conducted by a highly experienced doctor. If tendonitis is extensively present on the pulley, the release of the tendon sheath may be insufficient. Moreover, endeavoring to dissect the residual tendon sheath through the initial minor incision is likely to result in failure. In such cases, performing a repeated release from the same incision can result in minor skin lacerations, and extended incisions are inevitably required, which often preclude stable early postsurgical therapy.

Therefore, we have modified this surgical method by passing the guide knife through a 20-guage needle hole instead of using a scalpel skin incision, thereby making the technique less invasive. When one needle hole is insufficient to release the A1 pulley, another needle hole can be created to release the A1 pulley.

This technique of releasing the A1 pulley bilaterally through the eyes of a needle without the scalpel skin incision makes the use of water possible, which is essential for daily activities, in just 2 days after surgery. In this case report, we describe a patient with trigger finger who underwent successful percutaneous surgery, thereby enabling very early posttreatment rehabilitation.

## Case Report

A 61-year-old woman presented to another hospital because of snapping in her right ring finger within the past year. The patient received steroid injections, after which her symptoms resolved. She had a recurrence of snapping 3 months before presenting at our institution. Her symptoms gradually deteriorated, and 3 days before presentation, her ring finger was fixed in a bent position. She was forced to gently correct it with the opposite hand because of the fear of pain (Quinnell grade III: locking, only passively correctable).^5^

At her first visit, she exhibited tenderness on palpation just proximal to the metacarpophalangeal flexion crease of the fourth digit. A tender nodule was also found in this area. She could make a tight fist with her right hand, but the ring finger remained in the flexed position when she was asked to extend her fingers. An audible snap occurred when the finger was passively extended using the contralateral hand. Her pain-related functional status corresponded to a score of 6 on the visual analog scale (VAS), a 10-point scale in which 1 indicates no pain and 10 indicates most severe pain.”^6^ Proximal interphalangeal joint flexion contractures were not observed. She had no history of diabetes mellitus, rheumatoid arthritis, or renal failure and no specific family history.

Surgical findings were as follows: the measurements of the A1 pulley were 7.0 mm long and 1.5 mm thick using ultrasonography (SONIMAGE, HS1, 226ABBZX00051000, probe; stick probe L18-4 [4–18 Hz]; Konica Minolta). Local anesthesia (4 mL of 1% mepivacaine hydrochloride) was administered just above the distal end of the A1 pulley of the middle finger by using a 27-guage injection needle.

**Figure 2:**
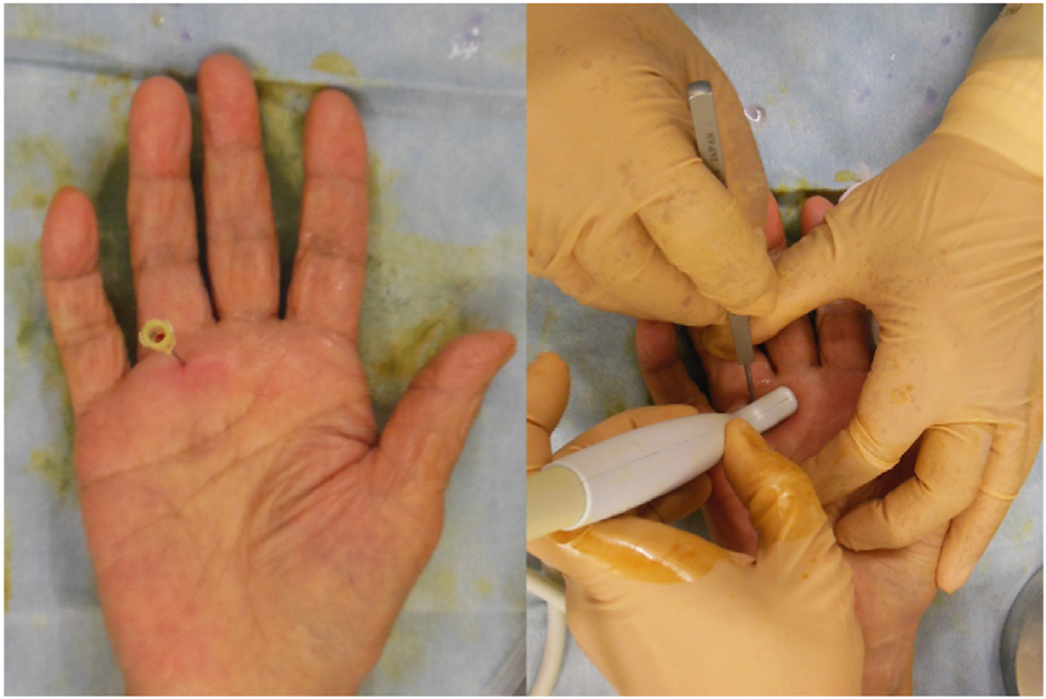
Placement of the 20-guage needle. The figure on the left shows a 20-guage needle inserted immediately above the proximal end of the A1 pulley. The figure on the right shows the guide knife inserted from the eye of the needle and its insertion checked using ultrasound.

**Figure 3:**
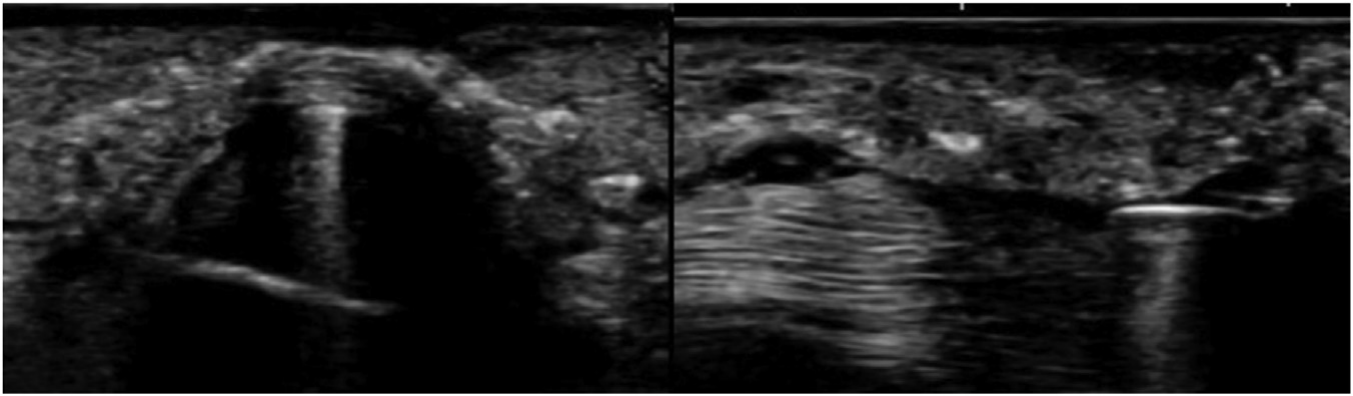
Ultrasonography within the tendon sheath. The left figure shows a short-axis ultrasound image. The guide within the tendon sheath and the neurovascular bundles on both sides of the tendon sheath are visible. The figure on the right shows the long-axis view. The guide is visible in the tendon sheath.

**Figure 4:**
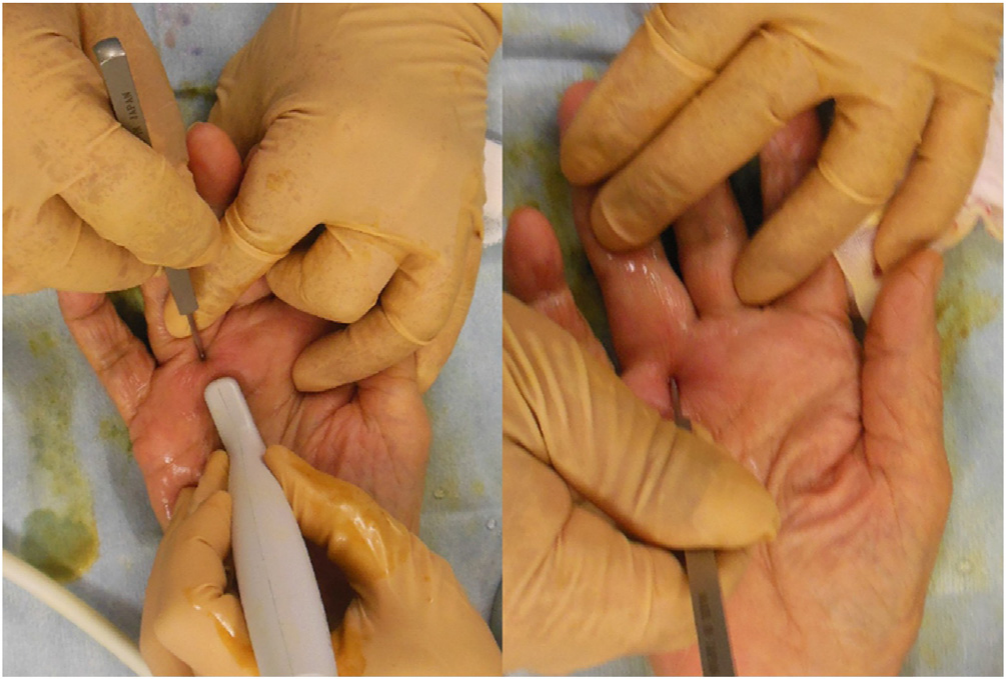
Incisions and pulleys. The figure on the left shows the thrust cut while checking the running of the tendon with ultrasonography after changing to a guide knife with a sharp blade. The figure on the right shows the release of the distal remnant tendon sheath of the A1 pulley and part of the proximal end of the A2 pulley after confirmation with ultrasonography.

A 20-guage needle was inserted at the same site as the local anesthetic, and a blunt Y-guided knife was inserted through the 20-guage needle hole, pointing proximally (Fig. 2). The guide was inserted into the tendon sheath and lifted under ultrasound to confirm that it had moved to the center just below the tendon sheath to prevent damage to the neurovascular bundles on both sides of the tendon sheath (Fig. 3). The guide knife was replaced with a sharp knife. The A1 pulley was released by thrusting it to the proximal end while flexing the metacarpophalangeal joint. The sharp guided knife was inserted distally through the same needle hole to release the thickened A1 remnant pulley and part of the proximal end of the A2 pulley (Fig. 4).

**Figure 5:**
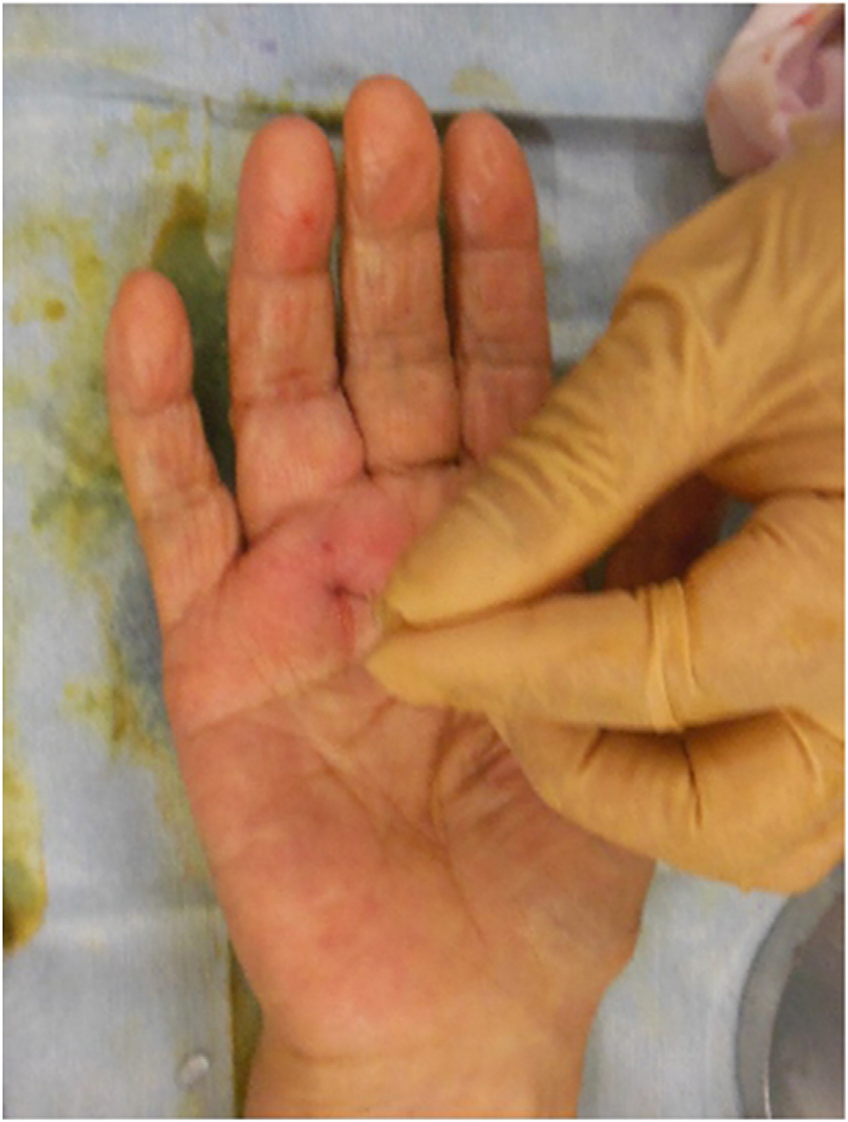
Creating the eye of the needle. The figure shows the creation of a needle in the second eye at the proximal end of the A1 pulley.

The snapping persisted; therefore, a second needle hole was created at the proximal end of the A1 pulley, and the same procedure was repeated (Fig. 5). The snapping disappeared, and the patient was able to bend and extend her ring finger smoothly. A moderate compression dressing was applied after surgery. For postoperative pain control, we prescribed 60 mg loxoprofen sodium three times a day for 3 days after surgery.

Two days after surgery, there were no complications, such as tendon and neurovascular injury or infection. Tendon movement was smooth, but there was mild pain (VAS score, 2) when waving the hand (Quinnell grade 0: normal movement, occasional pain).

**Figure 6:**
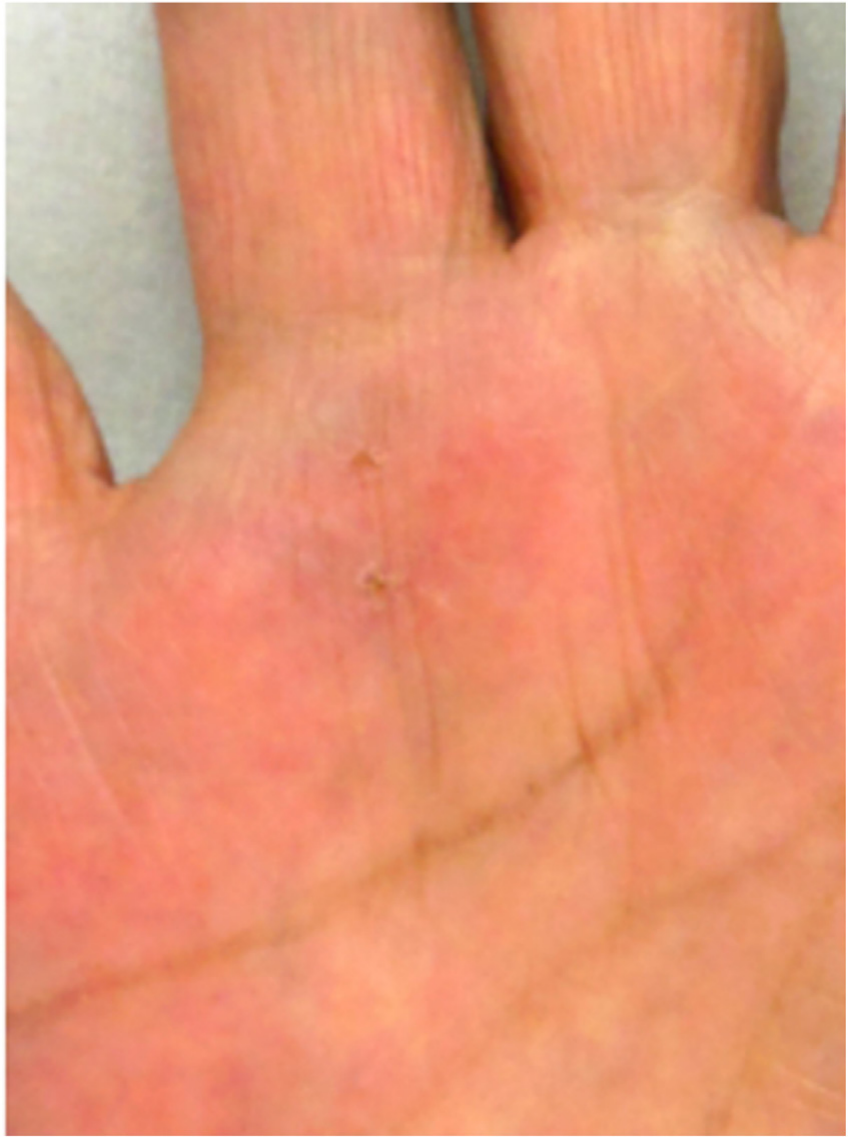
Healing at 1 week after surgery. The figure shows virtually no needle marks at 1 week after surgery.

The pinhole wounds were closed, and the patient was able to perform kitchen work. One week after the operation, there was slight scar formation in the needle holes but no associated interruption in the daily activities of the upper extremities (eg, eating, dressing, washing the face, and bathing)—except for twisting, for example, when opening a screw-thread lid of a bottle (rated as a VAS score of 1) (Fig. 6). One year after surgery, the pain-related functional status VAS score was 0 with no recurrence (Quinnell grade 0: normal movement, no pain). The patient’s degree of satisfaction was “excellent” in all postoperative evaluations.

### Informed consent

We verbally explained to the patient the disclosure of images, surgical findings, and follow-ups that did not allow personal information to be identified. Written consent was obtained.

## Discussion

We report a case in which a hockey stick–like guided knife was inserted through only two needle holes to release the A1 pulley safely and reliably. This novel technique, which does not involve a scalpel skin incision, is the least invasive to the soft tissue. As a result, the patient was able to return to daily life activities, such as kitchen work, in just 2 days without delaying posttreatment rehabilitation. This is especially notable as the complete release of the A1 pulley was extremely difficult for this patient.

A major disadvantage of conventional open surgery is the longer postoperative recovery period, which is approximately 2 weeks.^3^ However, this novel surgical technique is atraumatic for the skin and soft tissues.^4^ This percutaneous surgery consequently allows patients to return to daily life within a few days after surgery, even in difficult cases.

The knife used in this report (Y’s Guided Knife) was developed by Yumoto^4^ in 1970, and its clinical use began in 1989 when neuromusculoskeletal ultrasound was not widespread. Yumoto^4^ reported that the patients underwent 215 surgeries (139 thumbs and 76 other digits). Over a mean follow-up of 3.5 years, the clinical outcomes of patients treated with Y’s Guided Knife, including those with diabetes, included only two triggering recurrences and no complications, such as neurovascular damage or infection. Yumoto^4^ performed this surgical procedure with a small skin incision using a scalpel and reported it as safe and reliable.

We devised a percutaneous surgery in which Y’s Guided Knife passes through a needle instead of using a scalpel skin incision. By performing this surgical procedure through a few needle holes, the release of the tendon sheath becomes even less invasive, and the posttreatment recovery can be remarkably shortened for trigger finger in adults. Further randomized controlled trials with sufficient sample sizes are needed to evaluate recurrence, complications, postoperative pain relief, and proximal interphalangeal flexion contractures.

## References

[1] Yin L., Guo R. (2016). Application and progress of high frequency ultrasound in trigger finger. Chin J Med Ultrasound.

[2] Nikolaou V.S., Malahias M.A., Kaseta M.K., Sourlas I., Babis G.C. (2017). Comparative clinical study of ultrasound-guided A1 pulley release vs open surgical intervention in the treatment of trigger finger. World J Orthop.

[3] Huisstede B.M.A., Hoogvliet P., Coert J.H., Friden J., European HANDGUIDE Group (2014). Multidisciplinary consensus guideline for managing trigger finger: results from the European HANDGUIDE Study. Phys Ther.

[4] Yumoto Ortho Clinic Guided Knife. http://www.yumoto-ortho.jp/yoc-e-net-tmp/index.html.

[5] Langer D., Maeir A., Michailevich M., Luria S. (2017). Evaluating hand function in clients with trigger finger. Occup Ther Int.

[6] Breivik H., Borchgrevink P.C., Allen S.M. (2008). Assessment of pain. Br J Anaesth.

